# Performance of the procedure for ultra-rapid extraction and loop-mediated isothermal amplification (PURE-LAMP) method to detect malaria in Haiti

**DOI:** 10.1186/s40249-023-01097-w

**Published:** 2023-05-22

**Authors:** Jeanne Perpétue Vincent, Alexandre Valcena Existe, Kanako Komaki-Yasuda, Jacques Boncy, Shigeyuki Kano

**Affiliations:** 1 Department of Tropical Medicine and Malaria, Research Institute, National Center for Global Health and Medicine, Tokyo, 162-8655 Japan; 2 Graduate School of Comprehensive Human Sciences, University of Tsukuba, Tsukuba, 305-8575 Japan; 3 Laboratoire National de Santé Publique, 6120 Port-au-Prince, Haiti; 4 Present Address: Unité d’Épidémiologie des Maladies Émergentes, Institut Pasteur, Paris, France

**Keywords:** Diagnosis, Dried blood spots, Haiti, Loop-mediated isothermal amplification (LAMP), Low transmission, Malaria, Microscopy, Nested PCR, Procedure for ultra-rapid extraction (PURE), Rapid diagnostic tests

## Abstract

**Background:**

Malaria continues to cause burden in various parts of the world. Haiti, a Caribbean country, is among those aiming to eliminate malaria within a few years. Two surveys were conducted in Haiti during which we aimed to evaluate the performance of the simple and rapid procedure for ultra-rapid extraction–loop-mediated isothermal amplification (PURE-LAMP) method with dried blood spots as an alternative diagnostic method for malaria in the context of low to very low rates of transmission.

**Methods:**

Febrile and afebrile people were recruited from three administrative divisions within Haiti: Nippes, Sud and Grand’Anse, during the summers of 2017 (early August to early September) and 2018 (late July to late August). Their blood samples were tested by microscopy, rapid diagnostic tests (RDT), PURE-LAMP and nested PCR to detect *Plasmodium* infection. Sensitivity, specificity, positive and negative predictive values and kappa statistics were estimated with the nested PCR results as the gold standard.

**Results:**

Among 1074 samples analyzed, a positive rate of 8.3% was calculated based on the nested PCR results. Among febrile participants, the rates in 2017 and 2018 were 14.6% and 1.4%, respectively. Three positives were detected among 172 afebrile participants in 2018 by PURE-LAMP and nested PCR, and all three were from the same locality. There was no afebrile participants recruited in 2017. The PURE-LAMP, RDT and microscopy had respective sensitivities of 100%, 85.4% and 49.4%. All of the testing methods had specificities over 99%.

**Conclusions:**

This study confirmed the high performance of the PURE-LAMP method to detect *Plasmodium* infection with dried blood spots and recommends its use in targeted mass screening and treatment activities in low endemic areas of malaria.

## Background

The World Health Organization estimated 247 million cases and 691 thousand deaths of malaria worldwide in 2021. Some regional progress has been made from the E-2020 countries (identified as having the potential to be malaria free by 2020). China and El Salvador were certified as malaria free in 2021. Iran, and Malaysia have accomplished their goal of three consecutive years without indigenous cases. In the Americas, Paraguay and Argentina achieved malaria elimination [1, 2]. There remain 18 malaria-endemic countries and territories in the Americas, which includes only 2 in the Caribbean: the Dominican Republic and Haiti, on the sole island of Hispaniola on which malaria elimination is also considered feasible [3, 4]. *Plasmodium falciparum* is the endemic species, transmission is mainly focal, and the annual incidence was < 1% in Haiti and reached < 0.1% in 2018 whereas < 1000 cases were reported for the Dominican Republic [1, 5]. Both countries have a population of approximately 11 million inhabitants.

When transmission reaches a very low level, it is important to have sensitive tools that help detect the remaining reservoirs that need to be cleared to avoid the reestablishment of high positive rates within the population [6, 7]. Nucleic acid amplification techniques are the most accurate diagnostic choice [7]. Although polymerase chain reaction (PCR) assays to diagnose malaria have been developed since the 1990s, they remain centralized and too demanding for regular use in the endemic field [8]. The loop-mediated isothermal amplification method (LAMP) is becoming a formidable alternative [9–11]. LAMP is fast, robust and less expensive than PCR and, subsequently, is applicable in less well-equipped laboratories although the current cost per sample is still higher than ideal.

Among the different malaria LAMP assays developed, commercial kits have become available from two companies: the Loopamp™ MALARIA Pan/Pf Detection Kit (Eiken Chemical, Tokyo, Japan) [12–22] and the Illumigene Malaria LAMP (Meridian Bioscience Inc., Cincinnati, OH, USA) [23, 24]. The Loopamp™ MALARIA Pan/Pf Detection Kit has been evaluated with a significant number of samples in various studies conducted in both endemic [13, 15, 19–22] and non-endemic settings [12, 16], using whole blood [12, 21] or dried blood spots [15, 16], and coupled with diverse DNA extraction methods. One of these methods is the procedure for ultra-rapid extraction (PURE), a quick and simple DNA extraction method, also developed by Eiken Chemical to prepare DNA solution suitable for the LAMP reaction. We previously evaluated the accuracy of the combination of PURE and Loopamp™ MALARIA Pan/Pf (PURE-LAMP) methods on dried blood spots of suspected and confirmed cases of malaria imported to Japan [16]. The performance of this system was proved at a Japanese laboratory. However, the greater impact of a LAMP-based system is expected in malaria-endemic areas through its application in screening and confirmation of a malaria diagnostic or parasitic clearance. Thus, the present study aimed to evaluate the PURE-LAMP malaria detection method in the low transmission context of Haiti along with concurrent observation of the epidemiological situation.

## Methods

### Study sites

**Fig. 1:**
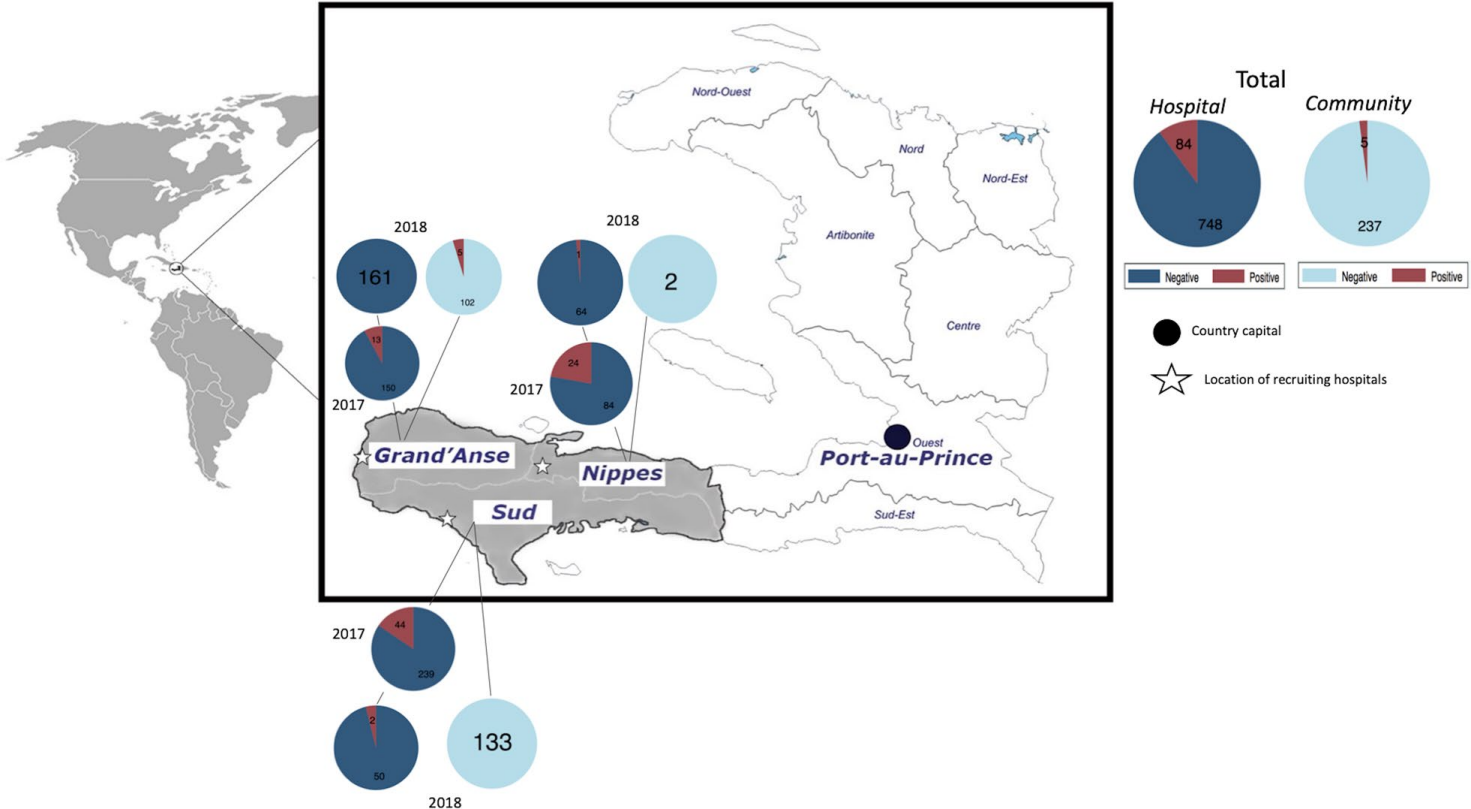
Location of the study area. Haiti is located in the Americas. The three departments (administrative divisions) of the study are highlighted in grey, and the others are shown in white. The number of participants by department, year and nested PCR diagnosis, is shown based on recruitment setting, hospital or community

Based on data from the national malaria control program, three departments (main administrative divisions in Haiti) with the highest positivity rates in 2016 and early 2017 were selected as study sites. They were Nippes, Sud, and Grand’Anse in the southwestern part of the country (Fig. 1), with respective annual parasite indexes of 2.5, 4.4 and 22.3 in 2016 and 2.9, 7.6, and 18.4 in 2017 per 1000 population. Sud and Grand’Anse shared 63.6% of the malaria cases registered in 2016 in the country. Although a descending or at least stable incidence trend has been registered in the other 7 departments since 2015, the three chosen departments have shown an increasing or stable trend. Recruitment was carried out in small coastal cities with less than 50,000 inhabitants and which were located remotely from the capital cities of each department. At the start of the study, the sequels of hurricane Matthew that had hit 10 months earlier (October 2016) could still be spotted in a recovering environment.

Light microscopy is dictated as the gold standard for malaria diagnosis by the Haitian Ministry of Health; however, after allowing the alternative use of rapid diagnostic tests (RDT) in recent years, they are more widely used [4, 25, 26]. Besides the availability of these tests and malaria treatment free of charge for patients, vector control activities had been carried out under the national malaria control program.

### Recruitment

People aged 1 year and over were recruited following two approaches: (i) hospital-based recruitment targeting participants with fever on the day of their hospital visit or anytime up to 2 weeks earlier, and (ii) community-based recruitment targeting the general population during social gatherings. There was no criterion for fever. Most social group participants were afebrile, but some people recruited there reported a history of fever.

Recruitment was carried out simultaneously at the same three health facilities, Centre de santé de Baradères (Nippes), Hôpital de Port-à-Piment (Sud) and Hôpital de la communauté de Dame-Marie (Grand’Anse), during the summers of 2017 (early August to early September) and 2018 (late July to late August). Community-based recruitment was carried out in Port-à-Piment, Port-Salut (Sud) and Petite Rivière de Dame-Marie. At the point of recruitment, participants had to submit a consent form, answers to a short questionnaire about demography, and their fever history and preventive practices, and venous blood samples were collected by a qualified staff member either in ethylenediaminetetraacetic acid (EDTA)- or heparin-containing blood tubes. After blood collection, the participants were tested immediately with an SD Bioline Malaria Ag Pf/Pan RDT (Standard Diagnostics, Inc., Suwon, the Republic of Korea) in accordance with the manufacturer’s guidelines, and blood smears were prepared and 100 μl of blood was seeded on filter paper (Whatman™ FTA™ classic cards; GE Healthcare, Tokyo, Japan) and air dried. After drying, each card was stored in a plastic bag containing a small desiccant bag. The participants were treated immediately if the RDT result was positive. Blood smears were fixed, stained and transported to the same laboratory along with the filter papers. The other tests (PURE-LAMP, PCR, microscopy) were conducted afterwards.

This study used venous blood to avoid double invasion of the participants as the majority seeking care were also predicted to require other blood tests. A small volume (100 μl) was used to prepare dried blood spots to approximate capillary blood sampling.

### Microscopy

Two microscopists based at a Haitian reference laboratory visualized thick and thin Giemsa-stained smears using a × 1000 light microscope for each participant. Three hundred microscopic fields of the thick smear were visualized before declaring a negative sample. Parasite density was determined as percentage of parasitized red blood cells after observing about 5000 of them using the thin smear. Any positive was seen by a third microscopist who also assessed 10% of the negatives. The microscopists were blinded to the other test results of the participants.

### PURE-LAMP

DNA was extracted with the Loopamp™ PURE DNA Extraction Kit from three dried blood spots with a diameter of 3 mm following the manufacturer’s guidelines and as reported elsewhere [16]. Reactions were carried in two tubes for all samples with the Loopamp™ MALARIA Pan/Pf Detection Kit, a Pan (targeting *Plasmodium* spp.) and a Pf (*P. falciparum*-specific) tube for each. Negative and positive controls (provided in the kit) were also included in each run.

The presence of amplified DNA inside the reaction tubes may be recognized in two speedy ways: (i) by fluorescence under UV excitation due to calcein unquenched during reactions, and (ii) by turbidity due to the precipitation of magnesium pyrophosphate [27]. In this study, only end-point evaluation by fluorescence under UV excitation was conducted for samples collected in heparin tubes giving a dichotomous positive or negative result; while real-time turbidity was measured with a Loopamp EXIA real-time turbidimeter (Eiken Chemical, Tokyo, Japan) for hospital samples collected in EDTA blood tubes, giving a turbidity threshold time (tt) for amplified samples. This was done to exclude any possibility of false positives due to EDTA chelating properties.

### DNA extraction for PCR

DNA samples were extracted from three dried blood spots with a diameter of 3 mm using a Maxwell® RSC DNA FFPE Kit (Promega, Madison, WI, USA) and the automated Maxwell® RSC Instrument (Promega, Madison, WI, USA). Minor modifications to the manufacturer’s guidelines can be found in a previous report [28]. DNA was eluted in 50 μl of elution buffer and stored at 4 °C.

### Nested PCR

The reference test in this study was a nested PCR system targeting the *Plasmodium* 18S ribosomal RNA gene [29]. This method generally implies a secondary step with species-specific primer sets for all human *Plasmodium*. However, secondary PCRs in this study were conducted only with a primer set specific to *P. falciparum*, which is the species expected in Haiti. For discrepant samples between PURE-LAMP ( +) and nested PCR (−), both tests were repeated, and the number of secondary PCR cycles was increased from 20 to 30. The nested PCR diagnosis was updated if a previously negative sample became positive at this step.

### Statistical analysis

Assuming 5% positivity rate among febrile participants, PURE-LAMP sensitivity of 97%, specificity of 99% [16], at least 895 participants samples had to be analyzed to estimate the performance of PURE-LAMP with a marginal error of 5% and confidence level of 95%.

Data analysis was performed with Stata (StataCorp, TX, USA). Sensitivity, specificity, positive predictive value and negative predictive value with their respective 95% confidence intervals (*CI*s) and kappa statistics were estimated with nested PCR as the reference.

## Results

### Characteristics of the study participants

In total, 1115 people gave consent to participate in this study: 265 in community settings and 850 at the hospitals. After exclusions due to missing samples, 554 blood samples were analyzed from those recruited during the summer of 2017, all from hospitals, and 520 from those of summer 2018, including 242 from community-based recruitment (Fig. 1). The participants ranged in age from 1 to 85 years old (mean: 31.0; *SD*: 20.5 years) and included 366 (34.7%) males and 690 (65.3%) females. Among them, 225 (21.1%) had a personal history of fever on the day of recruitment, 488 (45.7%) from a day before up to 7 days earlier and 183 (17.1%) from 8 days up to 2 weeks earlier, and 284 (26.9%) reported having a household member with fever and 92 (8.7%) a close neighbor. The history of having a relative with fever was significantly related to being tested positive for malaria by nested PCR or PURE-LAMP (*P*-value: 0.000). Preventive behaviors were quite common: 948 (88.3%) participants reported at least one, with the use of a bed net being the most reported (584 participants) (Table 1).

**Table 1:** Characteristics of the study participants by year and recent fever history

Characteristic	2017 Febrile	2018 Febrile	2018 Afebrile	Total (%)
Participants, *n* (%)	554 (51.6)	348 (32.4)	172 (16.0)	1074 (100)
Age (years), mean (*SD*)	27.4 (18.3)	29.1 (18.6)	46.4 (23.5)	31.0 (20.5)
Last episode of fever, *n* (%)			Not applicable	
Day 0	84 (15.3)	141 (40.8)		225 (21.1)
1–7 days	315 (57.2)	173 (50.0)		488 (45.7)
8–14 days	151 (27.4)	32 (9.2)		183 (17.1)
Known relative with fever, *n* (%)				
No	296 (54.5)	248 (71.5)	138 (82.6)	682 (64.5)
At home	173 (31.9)	91 (26.2)	20 (12.0)	284 (26.9)
Neighborhood	74 (13.6)	8 (2.3)	10 (6.0)	92 (8.7)
Preventive practices, *n* (%)				
Bed net	255 (48.2)	234 (70.5)	95 (78.5)	584 (59.5)
Insecticide (spray)	24 (4.5)	16 (4.8)	19 (15.7)	59 (6.0)
Window screen	2 (0.4)	0	0	2 (0.2)
Fumigation	215 (40.6)	91 (27.4)	32 (26.4)	338 (34.4)
Mosquito repellent	1 (0.2)	0	2 (1.7)	3 (0.3)
Chemoprophylaxis	1 (0.2)	0	0	1 (0.1)
Positive, *n* (%)				
Nested PCR	81 (14.6)	5 (1.4)	3 (1.7)	89 (8.3)
Microscopy	47 (8.5)	4 (1.1)	0	51 (4.7)
RDT Pan	54 (9.7)	2 (0.6)	0	56 (5.2)
RDT Pf	76 (13.7)	5 (1.4)	0	81 (7.5)
PURE-LAMP Pan	90 (16.2)	5 (1.4)	3 (1.7)	98 (9.1)
PURE-LAMP Pf	88 (15.9)	5 (1.4)	3 (1.7)	96 (8.9)

*RDT Pan* rapid diagnostic test band to detect *Plasmodium* lactodehydrogenase, *RDT Pf* band for detection of *P. falciparum-*specific histidine rich protein II, *PURE-LAMP* procedure for ultra-rapid extraction loop-mediated isothermal amplification, *Pan* tubes for diagnosis of *Plasmodium* genus, *Pf* *P. falciparum-*specific tubes

### Rates of positivity

The rates of positivity for malaria as estimated by each method and by year are summarized in Table 1. As concluded by nested PCR, there were 89 (8.3%) positives among 1074 samples that had been tested by all methods. The positive rate varied from 14.6% among samples collected in 2017 to 1.5% for samples from 2018. Although all participants in 2017 were febrile, the positive rate among febrile participants in 2018 was 1.4%. The positive rate among the afebrile participants was 1.7%. RDT and microscopy did not detect any *Plasmodium* infection among the afebrile participants. Microscopic parasitemia varied, ranging from 0.02% to 20.0% (mean: 2.3, *SD*: 3.2) when countable.

### Performance parameters

**Fig. 2:**
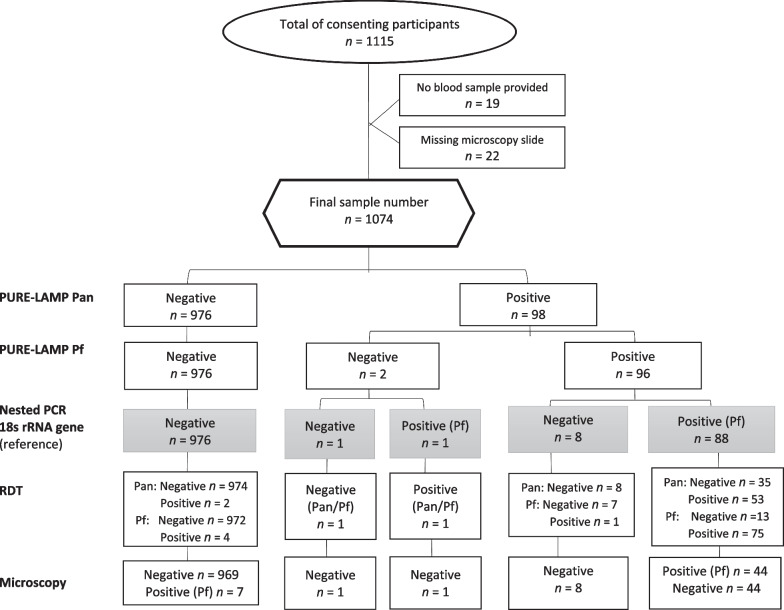
Flowchart of the study participants results by nested PCR, PURE-LAMP, RDT and microscopy. *PURE-LAMP* procedure for ultra-rapid extraction-loop-mediated isothermal amplification, *PCR* polymerase chain reaction, *Pan* tubes for diagnosis of *Plasmodium* genus, *Pf* *P. falciparum*-specific tubes, *RDT* rapid diagnostic test

An almost perfect agreement was calculated between the nested-PCR and PURE-LAMP (*Kappa*: 0.95). The PURE-LAMP Pan and PURE-LAMP Pf had respective sensitivities of 100% (95% *CI*: 95.9–100) and 98.9% (95% *CI*: 93.9–100), and their specificities were 99.1% (95% *CI*: 98.3–99.6) and 99.2% (95% *CI*: 98.4–99.6). The RDT Pf band had a sensitivity of 85.4% (95% *CI*: 76.3–92.0) and specificity of 99.5% (95% *CI*: 98.8–99.8), whereas for the RDT Pan band, values of 60.7% (95% *CI*: 49.7–70.9) and 99.8% (95% *CI*: 99.3–100), respectively, were calculated. Microscopy sensitivity was 49.4% (95% *CI*: 38.7–60.2), and its specificity was 99.3% (95% *CI*: 98.5–99.7) (Fig. 2 and Table 2).

**Table 2:** Performance of PURE-LAMP, RDT and microscopy against nested PCR as reference

	Sensitivity, n/d % (95% *CI*)	Specificity, n/d % (95% *CI)*	PPV, n/d % (95% *CI*)	NPV, n/d % (95% *CI*)	*Kappa*
PURE-LAMP Pan	89/89 100 (95.9–100)	976/985 99.1 (98.3–99.6)	89/98 90.8 (83.3–95.7)	976/976 100 (99.6–100)	0.95
PURE-LAMP Pf	88/89 98.9 (93.9–100)	977/985 99.2 (98.4–99.6)	88/96 91.7 (84.2–96.3)	977/978 99.9 (99.4–100)	0.95
RDT Pan	54/89 60.7 (49.7–70.9)	983/985 99.8 (99.3–100)	54/56 96.4 (87.7–99.6)	983/1018 96.6 (95.2–97.6)	0.73
RDT Pf	76/89 85.4 (76.3–92.0)	980/985 99.5 (98.8–99.8)	76/81 93.8 (86.2–98.0)	980/993 98.7 (97.8–99.3)	0.88
Microscopy	44/89 49.4 (38.7–60.2)	978/985 99.3 (98.5–99.7)	44/51 86.3 (73.7–94.3)	978/1023 95.6 (94.1–96.8)	0.60

*NPV* Negative predictive value, *PPV* Positive predictive value, *PURE-LAMP* procedure for ultra-rapid extraction–loop-mediated isothermal amplification, *Pan* tubes for diagnosis of *Plasmodium* genus, *Pf* *P. falciparum-*specific tubes, *RDT Pan* rapid diagnostic test band to detect *Plasmodium* lactodehydrogenase, *RDT Pf* band for detection of *P. falciparum-*specific histidine rich protein II, *n/d* numerator/denominator

## Discussion

The results of the present study showed high sensitivity and specificity for a malaria diagnosis by the PURE-LAMP method on dried blood spots in a setting of low *P. falciparum* transmission. There was an important decrease of the positivity rate between the two study periods. The reason for it might be varied including weather patterns or effective interventions.

Our previous evaluation of the PURE-LAMP method with dried blood spots conducted with imported cases in a non-endemic setting showed a sensitivity over 96% and specificity of 100% [16]. A review of previously reported evaluations of the Loopamp™ MALARIA Pan/Pf Detection Kit with > 35 samples per group in diverse settings showed sensitivity in a range from about 90% up to 100% and specificity from about 85% to 100% [12, 13, 15, 16, 21, 22]. The results of the present study also lie within these ranges.

In the present study, all discrepancies between the nested PCR and the PURE-LAMP Pan results were negative by the nested PCR and positive by the PURE-LAMP Pan; they were also negative by microscopy and mostly negative by the RDT (except for one RDT Pf-positive result). We think it is possible that some of these samples had very low amount of parasites DNA, as the precision is lower for samples with parasitemia around PCR’s limit of detection. Three samples became positive by nested PCR after repeating the test. A limit of detection of 0.01 parasites/μl had been found for this nested PCR with DNA extracted from 200 μl of blood, eluted in the same volume and starting with 2 μl of template [29]. The discrepant samples had tt values longer than the 15 min expected after the start of the reaction (Table 3) [27]. This could indicate a very low amount of the parasites’ DNA on the template, which required longer amplification time to produce enough magnesium pyrophosphate to be detected by the turbidimeter. However, we did not find a strong correlation between tt values and parasitemia in this dataset. A stricter experimental approach regarding blood sample volume and cell distribution will be necessary to determine whether this parameter can be helpful to estimate parasitemia.

**Table 3:** Discrepancies between PURE-LAMP and nested-PCR

Sample code	PURE-LAMP Pan (tt)	PURE-LAMP Pf (tt)
111	Positive (27.5)	Negative
174	Positive (16.2)	Positive (19.3)
234	Positive (21.5)	Positive (23.3)
242	Positive (22.5)	Positive (23.5)
277	Positive (19.1)	Positive (17.2)
704	Positive (18.3)	Positive (21.5)
707#	Positive (22.0)	Positive (18.1)
713	Positive (19.5)	Positive (20.5)
1310	Positive (20.2)	Positive (21.2)
276*	Positive (24.2)	Negative

*PURE-LAMP* procedure for ultra-rapid extraction-loop-mediated isothermal amplification, *PCR* polymerase chain reaction, *tt* turbidity threshold time, *Pan* tubes for diagnosis of *Plasmodium* genus, *Pf* *P. falciparum*-specific tubes

Average tt of PURE-LAMP Pan was 16.4 min (*SD*: 1.8; range: 13.1–24.2) among 82 concordant positives but 20.8 (*SD*: 3.2) min among 9 samples with PURE-LAMP Pan ( +) and nested PCR (-). All discrepant samples were negative by microscopy. # Denotes a sample positive by RDT Pf. * Denotes a sample positive by nested PCR and RDT. All other samples were negative by RDT and nested PCR

Some difference in the starting volume and extraction methods might have had some impact on the results. Although 3 DBS (~ 15 μl of blood) had been used for DNA extraction for LAMP by the PURE method and by Maxwell for nested PCR, there is inherent difference between the 2 methods. The PURE extraction renders about 120 μl of DNA solution of which 30 μl (corresponding to ~ 3 μl of blood) were used for each tube of LAMP reaction. From the 50 μl of DNA solution eluted at the end of the extraction by Maxwell, 2 μl were used for the primary PCR (corresponding to < 1 μl of blood). In this study we were interested in the PURE-LAMP system as diagnostic method due to its field-friendliness, not only to evaluate the LAMP kits. Thus, we had to use a different extraction method for PCR because the PURE is not validated for PCR.

RDT Pf band had a sensitivity over 85% and also agreed almost perfectly with the nested PCR results (*Kappa*: 0.88). Being among the three brands validated in Haiti [26]—the First Response Malaria Ag HRP2 (Premier Medical Corporation Ltd., Watchung, NJ, USA), CareStart Malaria HRP2 (Pf) (Access Bio, Inc., Monmouth Junction, NJ, USA) and SD Bioline- the RDT Pf band of the latter test was confirmed here to be an efficient option for the diagnosis of febrile cases. Rogier et al*.* evaluated the concordance between this RDT and a high-sensitivity (hs) RDT (Alere Malaria Ag P.f., Standard Diagnostics) in Haiti; they concluded that a hsRDT may have limited utility in a malaria setting like Haiti because of the minimal gain in sensitivity (0.2% more of the total population detected positive with the hsRDT compared to the conventional one) [30]. Here the gain in true positive by the PURE-LAMP Pan or the nested PCR represents 1.2% of the total population. However, as we consider the history of fever, only the PURE-LAMP and nested PCR allowed the detection of afebrile infections.

Among the RDT-negative samples, two were observed with microscopic parasitemia of > 0.6%, although microscopy had the lowest sensitivity in the present study. We considered the possibility of having some RDT Pf false negatives due to histidine rich protein 2 (*hrp2*) gene deletion, but *hrp2* and *3* could be amplified for those two samples and eight more that were negative by the RDT Pf band but positive by the nested PCR and the PURE-LAMP. Three more samples- RDT Pf (-), nested PCR and PURE-LAMP ( +)- could not be amplified for *hrp2*, nor two among them for *hrp3*. Other genes such as *pfcrt* and *k13* could not be amplified for those three samples either, so this does not prove deletion. Herman et al*.* reported no evidence of *hrp2/3* deletion in Haiti as well [31]. This result could have been caused by variation in antigen expression, blood concentration or a technical error.

Five RDT false-positives were also found in this study, one coinciding with a PURE-LAMP false-positive. RDT false-positive may be caused by antigen persistence in the blood after an episode of malaria. Those cases reported personal history of fever within 2 weeks of the recruitment. It is possible that their recent fever was due to malaria. We think that the false positives by microscopy were due to artifacts during slide coloration.

In general, a decrease of malaria prevalence was observed between 2017 and 2018 in Haiti [3, 5]. The impact of hurricane Matthew might also have influenced malaria transmission in 2017 whereas malaria control interventions might have had a better impact in 2018.

While the prevalence of infection was significantly decreasing in hospitals and was reported to the national control program, some malaria foci may require special intervention including a different method of testing. In Dame-Marie (Grand’Anse), absolutely no febrile patients were diagnosed as malaria positive during the weeks of hospital recruitment in 2018 (Fig. 1). However, when a single-day community recruitment effort was organized in a locality about 3 km away from the hospital, two positives were first detected by the RDT Pf band. The number of positives from that day increased to five after performing PURE-LAMP and nested PCR. The first two positives were people with history of fever, whereas the other three had no recent history of fever. It is not known whether the febrile patients who participated in the community screening had planned to visit the hospital. We may also note that despite free malaria care, in Haiti visiting even a public hospital generally requires a small fee which is supported by patients out of pocket. The locality in question was known by the regional staff for having a higher risk of malaria infection and seemed to correlate well with a hotspot as defined by Bousema et al. [32]. Still, the present study showed that the RDT and microscopy methods were not exposing an accurate picture of malaria positivity.

Several studies have highlighted the importance of asymptomatic infections for transmission in low-transmission settings [6, 33–35]. It was estimated that submicroscopic carriers are the source of 20–50% of all human-to-mosquito transmissions when transmission reaches very low levels [6]. The present study provides data about an eligible method that can help detect those submicroscopic carriers that may be used for mass or reactive screening for downstream treatment.

The cost of PURE-LAMP (USD 40 per sample) is surely a barrier for its adoption in Haiti although performant. We don't think it would be cost-effective for this system to replace RDT at all levels of care. However, its introduction in regional laboratories could benefit the malaria program. Dried blood spots can be prepared by finger pricking at any health facility, gathering place or home, and be shipped. We think this test is better applied in focused community screening and reactive screening in low-endemic settings. Our opinion is pended to a cost-effectiveness analysis.

Despite the difference observed in positive rates between the periods studied, the challenge to achieve malaria elimination is enormous. This small number of afebrile infections is important for the community, considering that submicroscopic infections may still be transmitted and that the possible worst effect will occur in non-immune people. In future molecular studies in Haiti, the distribution and significance of these afebrile infections need to be explored in a larger sample size.

## Conclusions

The present study confirmed the high performance of the PURE-LAMP method for malaria testing with dried blood spots. It allowed the detection of febrile cases and afebrile infections missed by the RDT and microscopy in a low-transmission setting. Malaria prevalence was found to be significantly lower in 2018 compared to 2017 across the study sites in Haiti; a specific focus of infection appeared to require intervention with sensitive malaria diagnostic methods and treatment. We recommend the use of this diagnostic system in targeted mass screening and treatment to detect the remaining human infectious reservoirs for their clearance as a means to accelerate the elimination of malaria.

## Data Availability

The dataset analyzed during the current study is available from the corresponding author on reasonable request.

## Abbreviations

EDTA: Ethylenediaminetetraacetic acid
HRP: Histidine rich protein
LAMP: Loop-mediated isothermal amplification
NPV: Negative predictive value
PCR: Polymerase chain reaction
PPV: Positive predictive value
PURE: Procedure for ultra-rapid extraction
RDT: Rapid diagnostic test
